# Evaluation of kit-based loop-mediated isothermal amplification (TB-LAMP) assay in the diagnosis of tubercular lymphadenitis

**DOI:** 10.1099/acmi.0.000665.v3

**Published:** 2023-11-09

**Authors:** Saurav Das, Neeraj Nischal, Binit Kumar Singh, Animesh Ray, Pankaj Jorwal, Manish Soneja, Maroof Ahmad Khan, Kapil Sikka, Sanjeev Sinha, Naveet Wig

**Affiliations:** ^1^​ Department of Medicine, All India Institute of Medical Sciences, New Delhi, India; ^2^​ Department of Biostatistics, All India Institute of Medical Sciences, New Delhi, India; ^3^​ Department of Otorhinolaryngology, All India Institute of Medical Sciences, New Delhi, India

**Keywords:** TB-LAMP, EPTB, LNTB, *Mycobacterium tuberculosis c*omplex, CRS (composite reference standard)

## Abstract

**Background.:**

The rapid and accurate diagnosis of tubercular lymphadenitis remains a challenging task today. The World Health Organization (WHO) endorsed the LoopAMP MTBC kit (TB-LAMP) as a replacement for sputum smear microscopy in the diagnosis of pulmonary tuberculosis (PTB). However, no prospective diagnostic accuracy study of TB-LAMP for tubercular lymphadenitis in adults has been performed yet. The current study evaluated the diagnostic performance of TB-LAMP in tubercular lymphadenitis (LNTB).

**Methods.:**

In a prospective observational study conducted at a tertiary care hospital in India, 90 subjects (age >18 years) suspected of LNTB were recruited consecutively and followed up for 6 months between January 2019 and December 2020. Samples were processed for microscopy, culture, GeneXpert, histopathology and TB-LAMP. The sensitivity, specificity, positive predictive value (PPV) and negative predictive value (NPV) of TB-LAMP against the composite reference standard (CRS) and culture were determined.

**Results.:**

TB-LAMP showed a sensitivity of 83.78 % (95 % CI, 73.76–90.47) and a specificity of 81.25 % (95 % CI, 56.99–93.41), respectively, against the CRS. The PPV and NPV were 95.38 % (95 % CI, 87.29–98.42) and 52.00 % (95 % CI, 33.50–69.97), respectively. TB-LAMP showed a sensitivity of 88.89 % (95 % CI, 71.94–96.15) and a specificity of 36.17 % (95 % CI, 23.97–50.46), respectively, against culture. The PPV and NPV were 44.44 % (95 % CI, 32–57.62) and 85 % (95 % CI, 63.96–94.76), respectively.

**Conclusion.:**

TB-LAMP can be used instead of conventional microscopy for the diagnosis of TB in lymph node specimens at primary healthcare centres. It provides rapid and cost-effective diagnosis of LNTB in resource-limited settings due to good sensitivity and NPV.

## Data Summary

The authors confirm that all experimental data and protocols have been provided within the article.

## Introduction

Tuberculosis (TB) has plagued humanity for a long time. In 1993, the World Health Organization (WHO) declared TB a global health emergency [1]. There were an estimated 10 million new cases of TB worldwide in 2019, with an estimated 1.4 million deaths. India had the largest share (26 %) of the estimated new cases, with 2.64 million new cases and 0.45 million deaths from TB in 2019. Previous studies have estimated extrapulmonary TB (EPTB) to constitute 15–20 % of the total TB cases and up to 50 % of HIV–TB coinfections [2]. Tubercular lymphadenitis (LNTB) constitutes ~35 % of total EPTB cases [3]. As per National TB Elimination Programme (NTEP) data [4], the incidence of LNTB in India was 30.8 per 100 000 population in 2013. LNTB is a local manifestation of underlying systemic disease [5]. Thus, prompt diagnosis with timely initiation of appropriate therapy is essential.

For the past few decades, acid-fast bacillus (AFB) smear microscopy has been the mainstay of diagnosis of pulmonary tuberculosis (PTB), especially in resource-poor settings, due to its low cost and rapid results [6]. However, it is observer-dependent and has poor sensitivity. TB diagnosis by culture requires a long incubation time of 6–8 weeks. Nucleic acid amplification tests (NAATs) such as polymerase chain reaction (PCR) aid in the rapid, specific and sensitive diagnosis of LNTB but require technical expertise and expensive equipment. The GeneXpert (Cepheid, Sunnyvale, CA, USA) assay is limited by cost and dependence on sophisticated equipment/software.

The search for a true point-of-care test to aid the rapid, accurate and low-cost diagnosis of EPTB in a peripheral setting with limited resources is still ongoing. One of the promising candidates to have emerged so far is LoopAMP (LAMP), another easy-to-use, isothermal NAAT that relies on strand displacement DNA synthesis performed by DNA polymerase with high strand displacement activity in an autocycled reaction using two inner and two outer primers that recognize a total of six distinct sequences on the target DNA [7, 8]. It provides visible results that can be read easily and performed even in peripheral centres and has already been used to detect severe acute respiratory syndrome (SARS), malaria, influenza and African trypanosomiasis. One such test is the LoopAMP MTBC kit (TB-LAMP; Eiken Chemical Company, Tokyo, Japan), a commercial kit employing TB-LAMP technology to detect *Mycobacterium tuberculosis* complex (MTBC) rapidly in samples. The WHO has recommended TB-LAMP as a replacement test for sputum smear microscopy to diagnose PTB in adults with clinical features suggestive of PTB and as a follow-on test to smear microscopy in adults with clinical features suggestive of PTB, especially in sputum smear-negative specimens with a high index of clinical suspicion [6]. No guidelines have been formulated yet for the use of TB-LAMP in the diagnosis of LNTB.

Culture, although still considered the gold standard for LNTB diagnosis, has been termed ‘an imperfect reference standard’ [9]. In the systematic review performed by Kohli *et al*. in 2018 evaluating the GeneXpert assay in EPTB diagnosis, they found that culture did not perform well as the reference standard for LNTB [10]. Imperfect reference standards may lead to the wrong classification of subjects as cases and non-cases in diagnostic accuracy studies. The concept of a composite reference standard (CRS), comprising the results of several tests such as AFB smear, culture, histopathology/cytopathology, clinical evaluation and treatment response, was introduced to solve this issue [11, 12]. Vadwai *et al.* also used a CRS in their diagnostic accuracy study of GeneXpert in EPTB samples [13]. However, a CRS might have reduced specificity, leading to the misclassification of non-cases as cases. This would lead to wrong estimation of the sensitivity of the diagnostic test being evaluated, which would be lower than the actual sensitivity. Thus, a study evaluating TB-LAMP with culture and CRS, both used as reference standards, would be most appropriate to obtain a credible range of sensitivity and specificity [9].

We conducted a prospective observational study to evaluate the diagnostic accuracy of TB-LAMP for detecting LNTB, compared with CRS and culture, and assess the interrater reliability between TB-LAMP and GeneXpert.

## Methods

### Study setting

**Fig. 1.**
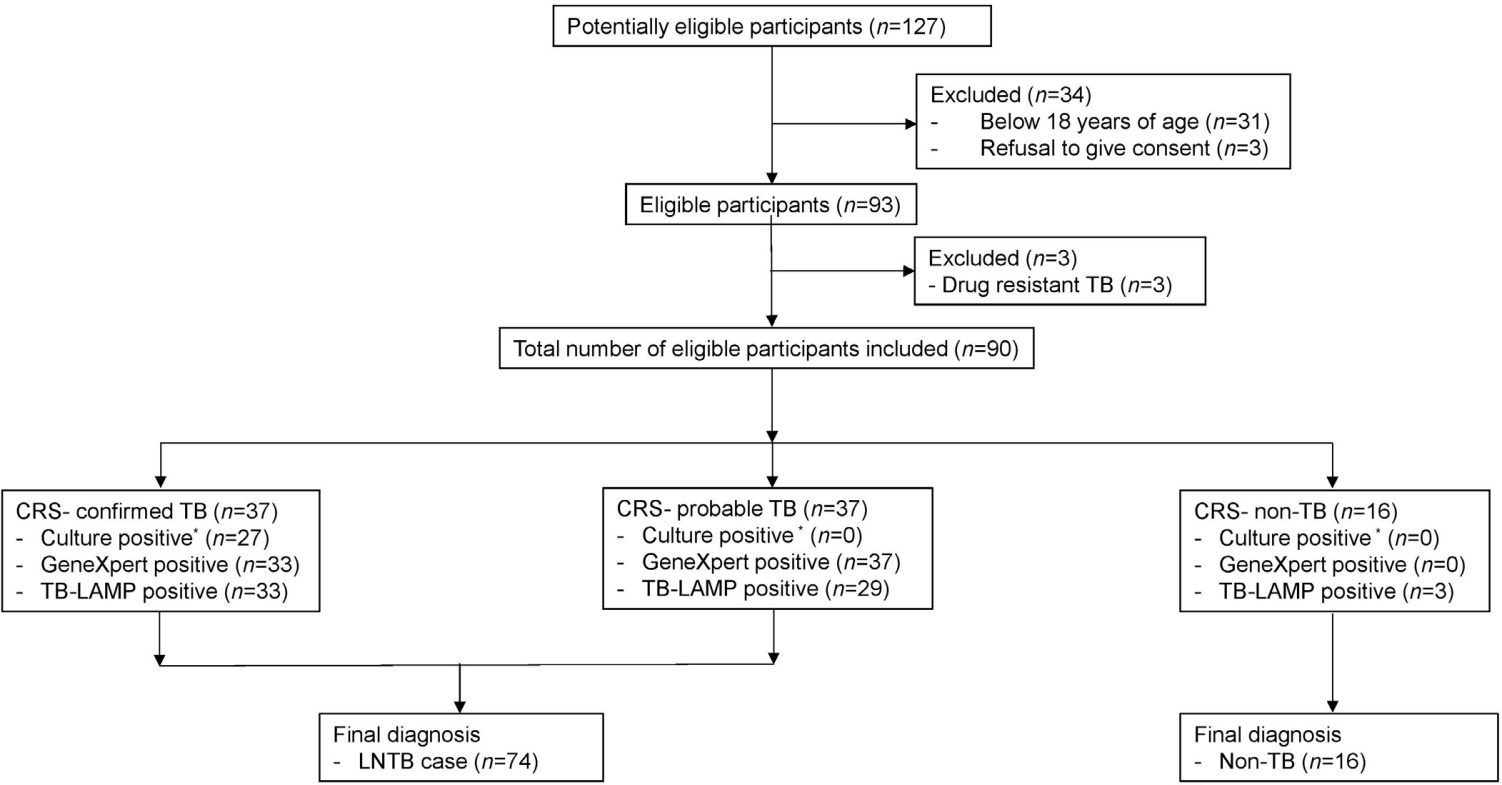
Recruitment of participants. ^*^Culture report unavailable for 3 CRS – confirmed TB, 10 CRS – probable TB and 3 CRS – non-TB cases. LNTB, tubercular lymphadenitis; CRS, composite reference standard; LAMP, loop-mediated isothermal amplification.

In this prospective observational diagnostic accuracy study, 90 patients (aged >18 years) were recruited consecutively from outpatient and inpatient settings, including the Infectious Diseases clinic and the medical ward. The treating physician advised during the routine investigations, after which the patients were screened and recruited after obtaining written informed consent. The study was conducted between January 2019 and December 2020 at All India Institute of Medical Sciences (AIIMS) New Delhi, India. A 6 month post-recruitment follow-up was conducted to assess the clinical response to anti-tubercular therapy (ATT) and the evolution of symptoms (Fig. 1).

### Sample size

We considered the sensitivity and specificity of AFB smear to be 76.47 and 100 %, respectively, while those of culture were 88.23 and 100 %, respectively, according to the reference study by Mittal *et al.* [14]. We also considered the sensitivity and specificity of GeneXpert in LNTB to be 81.20 and 99.10 %, respectively, according to the systematic review by Denkinger *et al.* [15]. At 95 % confidence interval and a margin of error of 7 %, and anticipating an increase in the sensitivity and specificity of CRS to 90 and 98 %, respectively (since CRS comprises AFB smear, culture and GeneXpert), the required number of cases and non-cases aweree 71 and 16, respectively.

### Inclusion criteria

Patients (>18 years of age) with clinical features compatible with tubercular lymphadenitis (LNTB) as per Index-TB guidelines [16], i.e. fever >7 days, X-ray/CT showing enlarged lymph nodes suggestive of TB, tender/non-tender enlarged lymph nodes, anorexia, weight loss, cough, shortness of breath, night sweats, dull or colicky pain, who were not on anti-tubercular therapy, were included.

### Exclusion criteria

Participants were excluded from the study if they refused to provide written informed consent, drug-resistant TB was detected or fine needle aspiration cytology (FNAC)/biopsy samples could not be obtained.

### Procedures

#### History and clinical examination

Patients attending the outpatient and inpatient settings were screened for symptoms compatible with LNTB, as mentioned in the inclusion criteria. History of symptom onset and duration and comorbidities, including diabetes mellitus, hypertension, HIV disease, previous history of TB, was taken. General physical examination of the enrolled study participants was performed in medical outpatient and inpatient settings, followed by a systemic examination (Table 1).

**Table 1.** Clinical characteristics

Characteristics		Total (*n*=90) *n* (%)
Mean age (years)		34±13.1
Male		50 (55.6 %)
Symptoms at presentation	Fever >7 days	84 (93.3 %)
	Weight loss	52 (57.8 %)
	Loss of appetite	44 (48.9 %)
	Cough	10 (11.1 %)
Site of lymphadenopathy	Cervical	43 (47.8 %)
	Mediastinal	20 (22.2 %)
	Axillary	11 (12.2 %)
	Supraclavicular	6 (6.7 %)
	Inguinal	2 (2.2 %)
	Cervical	43 (47.8 %)
Comorbidities	Previous history of tuberculosis	14 (15.6 %)
	Hypertension	4 (4.4 %)
	Diabetes mellitus	2 (2.2 %)
	HIV	5 (5.6 %)
Type of sample	FNAC	71 (78.9 %)
	Biopsy	19 (21.1 %)
Aetiology	Tuberculosis	74 (82.2)
	Reactive lymphadenopathy	6 (6.7 %)
	Metastasis	4 (4.4 %)
	Sarcoidosis	2 (2.2 %)
	Bacterial	1 (1.1 %)
	Kikuchi’s disease	1 (1.1 %)
	Hodgkin’s lymphoma	1 (1.1 %)
	Leukaemia	1 (1.1 %)

FNAC, fine-needle aspiration cytology;HIV, human immunodeficiency virus.

#### Haematological, biochemical and imaging investigations

These were performed as per the treating physician’s advice.

#### Investigations to detect *M. tuberculosis*

All microbiological lymph node samples (FNAC/biopsy in saline) were processed in the Intermediate Reference Laboratory, Department of Medicine, AIIMS, New Delhi, India. The samples were handled in a class 2 biosafety cabinet in a biosafety level 3 (BSL3) laboratory.

##### Sample processing and decontamination

Samples were initially processed and decontaminated by the standard N-acetyl-l-cysteine and sodium hydroxide (NALC–NaOH) method [17].

##### Smear microscopy

After decontamination, a smear was made on a glass slide using the resuspended pellet in phosphate-buffered saline (PBS), following which modified Ziehl–Neelsen (ZN) staining was performed as per the standard protocol [17], and microscopy was performed.

##### Culture

The decontaminated sample was used for inoculation in automated BACTEC MGIT 960 for liquid culture, following the standard protocol [17].

##### GeneXpert

The GeneXpert assay was performed according to the standard protocol [18].

##### TB-LAMP

The LoopAMP MTBC detection kit was used. First, 60 µl of the homogenized sample was transferred to a heating tube followed by heat inactivation at 95 °C for 10 min. The ultrarapid extraction (PURE) adsorbent tube was connected directly to the heating tube, and DNA extraction was completed. The PURE tube was assessed visually for the presence or absence of fluorescence from the reaction tube using UV light, after DNA amplification at 67 °C for 40 min, to determine the result according to the manufacturer’s instructions [19].

### Composite reference standard (CRS) and follow-up

Based on the clinical features, the results of histo-/cytopathological examination, AFB smear, MGIT culture, imaging or response to ATT, a CRS was devised that was similar to the one used by Vadwai *et al.* [13], and the participants were divided as follows.

- **Confirmed TB:** culture-/smear-positive with clinical features suggestive of TB.
- **Probable TB:** both culture and smear-negative, but:
  - GeneXpert-positive or
  - Histopathological examination or imaging compatible with TB along with clinical response to ATT present.
- **Non-TB:** culture and all other tests (GeneXpert, AFB staining, histopathology, imaging) for TB are negative, and the patient did not receive/respond to ATT.

For the final analysis, the confirmed and probable CRS categories were considered to be TB cases, while the non-TB CRS category was considered to be non-cases.

### Statistical analysis

The data were summarized and analysed using STATA version 14 software (StataCorp LLC, USA) to describe the patient’s demographics, clinical features, and examination and laboratory findings. Data were tested for normality using the Kolmogorov–Smirnov test. Data were expressed as mean+/−sd for normal distribution, median (IQR) or number and percentage as appropriate.

Sensitivity, specificity, negative predictive value (NPV), positive predictive value (PPV) and likelihood ratio were calculated to assess the diagnostic accuracy of TB-LAMP against CRS and MGIT culture; 95 % confidence intervals were calculated using the Wilson score interval. Agreement between TB-LAMP and GeneXpert in LNTB was calculated using Cohen’s kappa statistic. A *P*-value <0.05 was considered statistically significant.

## Results

Ninety participants with lymphadenopathy meeting the inclusion criteria were enrolled in the study, and a month follow-up was performed after enrolment, as per the study design.

There were 16 non-cases and 74 cases. Out of the 74 cases, 37 were in the confirmed TB and 37 in the probable TB category.

Out of the 16 non-TB subjects, 13 had a culture-negative report. A culture report was unavailable for the remaining three non-TB cases, and they were classified as non-TB cases based on the absence of response to ATT. All of the 16 non-TB subjects had a negative AFB smear.

Out of the 37 confirmed TB cases, 27 were identified based on a positive culture report. Out of the 27 TB cases with positive culture reports, only 12 were AFB stain-positive. Of the remaining 10 confirmed TB cases identified based on positive AFB staining, 7 had a negative culture report, and 3 did not have a culture report. Of the 37 probable TB cases, 27 had both negative culture and AFB smear reports, but a positive GeneXpert report and clinical response to ATT. The remaining 10 probable TB cases did not have a culture report and had a negative AFB smear, but had a positive GeneXpert report and a clinical response to ATT (Fig. 1).

### Diagnostic performance of TB-LAMP compared with CRS

Out of the 90 samples, 65 were positive on TB-LAMP. TB-LAMP showed a sensitivity of 83.78 % (95 % CI, 73.76–90.47) and a specificity of 81.25 % (95 % CI, 56.99–93.41), respectively, against CRS, as shown in Table 2. The PPV and NPV was 95.38 % (95 % CI, 87.29–98.42) and 52 % (95 % CI, 33.5–69.97), respectively. The likelihood ratios for a positive and negative test were 4.468 (95 % CI, 2.311–8.641) and 0.199 (95 % CI, 0.164–0.243), respectively.

**Table 2.** Comparison of TB-LAMP against CRS (*n*=90) and against MGIT culture (*n*=74)

		CRS (*n*=90)			Sensitivity (95 % CI)	Specificity (95 % CI)	PPV (95 % CI)	NPV (95 % CI)
		Positive	Negative	Total				
**TB –LAMP**	**Positive**	62 (68.9 %)	3 (3.3 %)	65 (72.2 %)	83.78 % (73.76–90.47)	81.25 % (56.99–93.41)	95.38 % (87.29–98.42)	52 % (33.5–69.97)
	**Negative**	12 (13.3 %)	13 (14.4 %)	25 (27.8 %)				
	**Total**	74 (82.2 %)	16 (17.8 %)	90 (100 %)				
		**MGIT culture (*n*=74)**			**Sensitivity** **(95 % CI)**	**Specificity** **(95 % CI)**	**PPV** **(95 % CI)**	**NPV** **(95 % CI)**
		**Positive**	**Negative**	**Total**				
**TB –LAMP**	**Positive**	24 (32.5 %)	30 (40.5 %)	54 (73 %)	88.89 % (71.94–96.15)	36.17 % (23.97–50.46)	44.44 % (32–57.62)	85 % (63.96–94.76)
	**Negative**	3 (4 %)	17 (23 %)	20 (27 %)				
	**Total**	27 (36.5 %)	47 (63.5 %)	90 (100 %)				

A culture report was unavailable for 16 participants (3 CRS – confirmed TB, 10 CRS – probable TB and 3 CRS – non-TB cases).

CI, confidence interval; CRS, composite reference standard; LAMP, loop-mediated isothermal amplification; NPV, negative predictive value; PPV, positive predictive value; TB, tuberculosis.

### Diagnostic performance of TB-LAMP compared with MGIT culture

TB-LAMP showed a sensitivity of 88.89 % and a specificity of 36.17 %, respectively, against culture, as shown in Table 2. The PPV and NPV were 44.44 % (95 % CI, 32–57.62) and 85 % (95 % CI, 63.96–94.76), respectively. The positive and negative test likelihood ratios were 1.393 (95 % CI, 1.291–1.502) and 0.307 (95 % CI, 0.130–0.724), respectively.

### Agreement between GeneXpert and TB-LAMP

The kappa statistic for agreement between TB-LAMP and GeneXpert was 0.44 (95 % CI, 0.23–0.65), which signifies a moderate agreement between the results of GeneXpert and TB-LAMP, as shown in Table 3.

**Table 3.** Agreement between TB-LAMP and GeneXpert(*n*=90)

		GeneXpert			Kappa statistic for agreement (95 % CI)
		Positive	Negative	Total	
**TB-LAMP**	**Positive**	58 (64.4 %)	7 (7.8 %)	65 (72.2 %)	0.44 (0.23–0.65)
	**Negative**	12 (13.3 %)	13 (14.5 %)	25 (27.8 %)	
	**Total**	70 (77.7 %)	20 (22.3 %)	90 (100.0 %)	

TB, tuberculosis; LAMP, loop-mediated isothermal amplification.

## Discussion

We conducted a prospective observational study including 90 study participants with a clinical suspicion of LNTB, out of which 74 were cases and 16 were non-cases. The observation of a higher specificity of TB-LAMP compared to CRS instead of culture can be explained by the low sensitivity of culture in LNTB. Further, the decontamination practices using NALC–NaOH can further decrease viable bacilli in LNTB, a paucibacillary form of TB [16]. False-positive TB-LAMP results can also be due to detection of dead bacilli and exposure to high humidity leading to non-specific false-positives, reducing the specificity of TB-LAMP.

In our study, the culture positivity rate was 36.40 % (27 positive reports out of 74 samples in TB cases). All 16 participants did not have a valid culture report due to an inadequate sample having been obtained. In comparison, the culture positivity rate was 15 % in the study by Sharma *et al.* [20], 15 % in the study by Mishra *et al.* [21] and 60 % in the study by Mittal *et al.* [14]. Culture showed a sensitivity of 50 % (95 % CI, 37.11–62.89) and a specificity of 100 % (95 % CI, 77.19–100), respectively, against CRS. The PPV and NPV were 100 % (95 % CI, 87.54–100) and 32.5 % (95 % CI, 20.08–47.98), respectively. Similar values were reported in the studies by Vadwai *et al.* [13] and Mittal *et al.* [14].The low sensitivity of culture can be attributed to the paucibacillary nature of LNTB and the decontamination techniques used for processing samples, which might further decrease the viable bacilli number, as shown by Sharma *et al.* in their study [16]. Wide-ranging values for culture sensitivity in the various studies and the low sensitivity in most studies have cast doubt on the use of culture as the sole reference standard in evaluating diagnostic tests in paucibacillary TB such as LNTB.

The level of statistical agreement between the results of TB-LAMP and GeneXpert was moderate; Cohen’s kappa statistic of 0.44 (95 % CI, 0.23–0.65). In our study, GeneXpert had a sensitivity of 94.59 % (95 % CI, 86.91–97.88) and a specificity of 100 % (95 % CI, 80.64–100) against CRS, respectively. Thus, GeneXpert was superior to TB-LAMP for detecting LNTB with the additional benefit of rifampicin resistance detection and estimation of bacillary load. The discrepancy between GeneXpert and TB-LAMP results could be due to the presence of inhibitors of DNA replication affecting GeneXpert, but not TB-LAMP. Simultaneously, the different DNA extraction kit provided by the manufacturer may be the other cause of the discrepancy between these diagnostics modalities.

The major limit of the present study is a slightly higher but acceptable margin of error of 7 % that had to be employed in our study with its small sample size due to logistical and cost constraints. Multidrug-resistant TB and rifampicin-resistant cases on GeneXpert were excluded from our study. It should also not be possible to replace rapid molecular tests with higher sensitivity for MTBC along with DR-TB detection. The LAMP assay used in the study is a commercial assay for the detection of MTBC. The difference in our results compared to previous studies can also be explained by the difference in genes targeted by the LAMP assays used, as shown in Table 4 [18, 22, 23]. While the TB-LAMP kit used in our study targets IS6110 and *gyrB,* the in-house LAMP assay used by Sharma *et al.* [16] targeted IS6110 and MPB64. Combinations of primers targeting various genes should be further explored to obtain a test with the best diagnostic accuracy. The study was conducted in a tertiary care hospital, while TB-LAMP is intended to be used for the diagnosis of MTBC as a point-of-care test in peripheral or rural settings instead of conventional microscopy, where results may be influenced by lower bacillary load and inappropriate sample collection and processing in the case of EPTB specimens.

**Table 4.** Review of of the LAMP literature for LNTB samples

Sl no.	Author	Study area	Sample size	Key findings
1	Our study	AIIMS, New Delhi	90 lymph node samples	The sensitivity, specificity, PPV, and NPV of LAMP against CRS were 83.78, 81.25, 95.38 and 52 %, respectively. Sensitivity, specificity, PPV and NPV of LAMP against culture were 88.89, 36.17, 44.44 and 85 %, respectively
2	Mishra *et al.* ^.^[18]	AIIMS Bhubaneswar	40 lymph node samples	The sensitivity, specificity, PPV and NPV of LAMP assay against culture were 33.3, 91.2, 40 and 88.57 %
3	Sharma *et al.* [17]	PGIMER, Chandigarh	170 lymph node samples (85 confirmed, 35 suspected and 50 control cases)	LAMP assay targeting both MPB64 and IS6110 had a sensitivity of 90 % and specificity of 100 % against CRS in LNTB diagnosis
4	Singh *et al.* ^.^[23]		100 EPTB samples	LAMP had a sensitivity of 85.71 % and specificity of 88.89 % of LAMP against culture

LNTB, tubercular lymphadenitis; CRS, composite reference standard; LAMP, loop-mediated isothermal amplification; CRS, composite reference standard; PPV, positive predictive value; NPV, negative predictive value; CI, confidence interval.

### Conclusion

Given its sensitivity of 84–89 % and specificity of 36–81 % compared to culture and CRS and its relative ease of use in peripheral settings due to there being no requirement for thermal cycling or sophisticated equipment, in addition to its shorter turnaround time, TB-LAMP may play a role in the diagnosis of MTBC as a point-of-care test for rapid and cost-effective diagnosis of LNTB in resource-limited, peripheral settings. However, TB-LAMP does not give any information regarding drug resistance or bacillary load, which is a drawback in comparison to GeneXpert, which can detect rifampicin resistance by detecting mutations in the *rpoB* gene. Thus, it can be used as the initial diagnostic as well as a follow-up test instead of smear microscopy, followed by referral to higher centres for mycobacterial culture and GeneXpert. Combinations of primers targeting various genes should be explored further to obtain a test with the best diagnostic accuracy.
